# Anxiety among Chinese primary school teachers under the “double reduction” policy: prevalence and associated factors

**DOI:** 10.3389/fpsyt.2026.1792092

**Published:** 2026-04-17

**Authors:** Xing Wang, Zeyun Hu, Yunhui Zhong, Baiping Tao

**Affiliations:** 1 Department of Neurosis and Psychosomatic Diseases, Huzhou Third Municipal Hospital, The Affiliated Hospital of Huzhou University, Huzhou, China; 2 Zhejiang Chinese Medical University, Hangzhou, China; 3 The Third People’s Hospital of Ganzhou, Ganzhou, China

**Keywords:** anxiety, double reduction, perceived stress, prevalence, primary school teachers

## Abstract

**Objectives:**

The implementation of the “Double Reduction” Policy in China has raised concerns about increased anxiety levels among primary school teachers. However, the prevalence of anxiety symptoms and the factors associated with them remain unclear. This study aims to investigate the prevalence and correlates of anxiety among primary school teachers under this policy.

**Methods:**

A cross-sectional survey was conducted from September to October 2022 among primary school teachers in 15 cities across China. Participants completed a series of questionnaires administered via WeChat to assess anxiety symptoms and potential related factors, including perceived stress, sociodemographic characteristics, and job-related variables. Anxiety and perceived stress were measured using the Generalized Anxiety Disorder-7 (GAD-7) and 10-item Perceived Stress Scale (PSS-10), respectively.

**Results:**

Overall, 44.5% (1,423/3,199) of teachers reported at least mild anxiety symptoms. Among them, 56.43%, 25.30% and 18.27% had mild, moderate, and severe anxiety, respectively. In the final logistic regression model, five variables remained independently associated with anxiety: education level (master’s degree or above: OR = 2.781, 95% CI: 1.858–4.163), income dissatisfaction (OR = 1.487, 95% CI: 1.205–1.834), intermediate professional title (OR = 1.372, 95% CI: 1.084–1.738), younger age (OR = 0.979, 95% CI: 0.966–0.993), and perceived stress (OR = 1.489, 95% CI: 1.443–1.538).

**Conclusion:**

Anxiety was prevalent among primary school teachers during the implementation of the “Double Reduction” Policy. Higher education level, income dissatisfaction, intermediate professional title, younger age, and elevated perceived stress were significant risk factors for anxiety. Therefore, interventions focusing on stress management and occupational support may help improve teachers’ mental well-being.

## Introduction

Teaching is a demanding profession, as teachers often report experiencing poorer mental health compared to other professionals (1, 2). Several studies have demonstrated that approximately 8% of teachers leave the profession (3), with a significant percentage (40%-50%) leaving within the first 5 years of their careers (4). Nayak defined anxiety as “a feeling of fear, worry, and uneasiness, usually generalized and unfocused as an overreaction to a situation that is only subjectively seen as threatening” (5). Anxiety among teachers is a global public health concern and has been associated with perceived stress. Research has indicated that a range of 13.67%-67.5% of teachers have reported experiencing anxiety (6–9). A recent large-scale study in China reported that 13.67% of the 88,611 teachers surveyed (94.75% response rate) across three cities in Henan Province experienced symptoms of anxiety (7). Anxiety can result in adversely affect not only on teachers’ mental health (10), but also on job performance (11).

The Chinese government implemented the “Double Reduction” Policy for compulsory education in July 2021, aiming to alleviate the burden of excessive homework and after-school tutoring (12). The goal was to achieve a significant reduction within one year and make remarkable progress within three years. Initial evidence suggests that the policy has begun to yield positive results (13, 14). For instance, a study investigated the changing trends in depressive and anxiety symptoms among 28398 Chinese elementary and junior high school students before and after the implementation of the “Double Reduction” Policy. The findings indicated a significant decrease in overall depression and anxiety levels, and a slight decrease in insomnia symptoms following the enactment of the “Double Reduction” Policy (15, 16).

Teachers play a crucial frontline role in implementing the “Double Reduction” Policy, however, it has not received adequate attention. Scholars have been focused on analyzing the implementation strategies and effects of the “Double Reduction” Policy from the perspective of students and parents, while overlooking the important role and status of teachers as participants in the same. This oversight inevitably imposes certain limitations on the research. A grounded theory study revealed that in-service primary and junior high school teachers held differing interpretations of the “Double Reduction” Policy, which had a certain impact on their anxiety levels. The rapid and complex nature of educational reform made teachers particularly susceptible to occupational anxiety (17). A study conducted by Jun Teng (18) and his colleagues also revealed that the implementation of the “Double Reduction” Policy has increased the working hours of Chinese primary school teachers, which, with heavy workloads, has adversely affected teachers’ well-being. Primary school teachers were selected as the focus of this study because they play a frontline role in implementing the “Double Reduction” Policy in daily educational practice. In China, primary school teachers are directly involved in policy-related tasks such as homework adjustment, after-school service provision, classroom management, and communication with parents. These responsibilities may increase their workload and emotional demands during policy implementation. In addition, previous studies have highlighted primary school teacher well-being as an important area of research, and emerging evidence suggests that the “Double Reduction” Policy may increase workload and working hours among Chinese primary school teachers (19). Therefore, focusing on this group may help to better understand the mental health challenges faced by teachers during educational reform.

Evidence remains limited regarding the relationship between perceived stress and anxiety among Chinese primary school teachers during the implementation of the “Double Reduction” Policy. Existing studies have mainly focused on teachers’ occupational anxiety under this policy context or on policy-related changes in teachers’ workload and working time, rather than specifically examining how perceived stress is associated with anxiety in primary school teachers (20). Therefore, this study aimed to: (1) estimate the prevalence of anxiety symptoms among Chinese primary school teachers during the implementation of the “Double Reduction” Policy; (2) compare demographic characteristics, job-related factors, and perceived stress between teachers with and without anxiety symptoms; and (3) identify factors independently associated with anxiety symptoms in this population.

## Methods

### Study design and participants

The present study follows the guidelines of the Strengthening the Reporting of Observational Studies in Epidemiology (STROBE) Statement checklist of items that should be included in reports of cross-sectional studies (21). A total of 3199 questionnaires were collected in this cross-sectional study. The participants consisted of primary school teachers from 15 cities across Southern China. To prevent duplicate submissions, each IP address or account was only allowed to submit the questionnaire once.

### Research instruments

#### Basic information

Sociodemographic and work-related information was collected, encompassing age, sex, height, weight, educational background, fertility situation (none/one child/two children/three children), years of teaching, income satisfaction (yes/no), marital status (single, married, divorced or widowed), school type (private school, public school), school location (urban/rural), whether serving as a class adviser (yes/no), and professional qualifications.

#### The generalized anxiety disorder-7 Scale

The GAD-7 scale is an internationally recognized seven-item self-assessment scale used to evaluate the severity of generalized anxiety disorder (22). Each item on the scale is scored on a 0-3 point Likert scale (3=“almost every day” and 0=“not at all”). The scores on the GAD-7 scale range from 0 to 21. The Chinese version of the GAD-7 scale has been widely utilized in China (23, 24). The Cronbach’s α coefficient of the GAD-7 scale was 0.87 in our study. The severity of anxiety symptoms on the GAD-7 is commonly classified into four categories according to score ranges: 0-4 (minimal), 5-9 (mild), 10-14 (moderate), and 15 or above (severe) (25). In our study, the participants were evaluated as having at least mild anxiety symptoms when the total scores ≥ 5 on the Generalized Anxiety Disorder-7 (GAD-7) scale. Therefore, the study categorized all subjects into an anxiety group and a non-anxiety group.

#### The 10-item perceived stress scale

The PSS was used to assess an individual’s perceived stress levels in terms of unpredictability and overload (26). Each item is rated between 0 and 4 points using a Likert scale (4=“very often” and 0=“never”). Six out of the 10 items assess the frequency of negative thoughts, whereas the remaining items evaluate the frequency of positive thoughts. The scores for all items are added up to calculate the total score, with the four positive items being reverse scored. Higher scores indicate a greater perception of psychological stress. The Chinese version of the scale demonstrates good reliability and validity (27, 28). In the present study, the Cronbach’s alpha value was 0.81 for the PSS.

### Research procedure

An online questionnaire was created and distributed via WeChat, one of the most important social tools in mainland China. Data were collected over 48 days, from September 14 to October 31, 2022.

### Ethical statement

This study was reviewed and approved by the Institutional Review Board (IRB) of the Third People’s Hospital of Ganzhou (Approved No. gzsyy2022029). All participants provided electronic informed consent after being briefed on the study’s purpose and procedures. Participant information was kept strictly confidential, and all procedures complied with the ethical standards of the Declaration of Helsinki.

### Statistical analysis

The normality of continuous variables was examined using the Kolmogorov–Smirnov test, and all variables were found to be normally distributed. Chi-square tests and analysis of variance (ANOVA) were used to compare sociodemographic, work-related, and perceived stress variables between the anxiety and non-anxiety groups. Variables that showed significant differences in the univariate analyses were subsequently entered into a binary logistic regression model using the backward stepwise method to identify independent factors associated with anxiety. All analyses were performed using SPSS version 26.0. A two-tailed p-value < 0.05 was considered statistically significant.

## Results

### Demographic characteristics of participants

A total of 3199 primary school teachers from 15 cities across China participated in the survey. Among all the participants, 1596 (49.9%) were male and 1603 (50.1%) were female. The age of participants ranged from 22 to 69 years old, with an average age of 39.51 ± 10.37 years. The average BMI was 23.23 ± 6.81 kg/m^2^. Among all the participants, 61.1% were dissatisfied with income. More detailed information about the demographic and job-related characteristics of the participants is listed in **Table 1**.

**Table 1:** Comparison of stress correlates between participants with and without anxiety.

Variable	Total	Non-anxiety	Anxiety	F/χ^2^	*P*-
	N=3199	(n=1776, 55.5%)	(n=1423, 44.5%)		
Age	39.51 ± 10.37	40.71 ± 10.90	38.02 ± 9.46	54.035	<0.001
Years of teaching	18.06 ± 11.80	19.41 ± 12.24	16.38 ± 11.02	52.616	<0.001
Sex				0.863	0.353
Male	1596 (49.9%)	873 (49.16%)	72 3(50.81%)		
Female	1603 (50.1%)	903 (50.84%)	700 (49.19%)		
Education				59.436	<0.001
Junior college or less	620 (19.4%)	402 (22.64%)	218 (15.32%)		
Bachelor	1985 (62.1%)	1118 (62.95%)	867 (60.93%)		
Master or above	594 (18.5%)	256 (14.41%)	338 (23.75%)		
Marital status				5.880	0.053
Never married	440 (13.8%)	243 (13.68%)	197 (13.84%)		
Married	2545 (79.6%)	1431 (80.57%)	1114 (78.29%)		
Divorced or Widowed	214 (6.6%)	102 (5.75%)	112 (7.87%)		
Fertility situation				13.206	0.004
None	563 (17.6%)	319 (17.96%)	244 (17.15%)		
One child	1492 (46.6%)	866 (48.76%)	626 (43.99%)		
Two children	968 (30.3%)	509 (28.66%)	459 (32.26%)		
Three children	176 (5.5%)	82 (4.62%)	94 (6.60%)		
Income satisfaction				149.945	<0.001
Yes	1245 (38.9%)	859 (48.37%)	386 (27.23%)		
No	1954 (61.1%)	917 (51.63%)	1037 (72.77%)		
Residence of school				3.459	0.063
Urban	1202 (37.6%)	642 (36.15%)	560 (39.35%)		
Rural	1997 (62.4%)	1134 (63.85%)	863 (60.65%)		
Teacher in charge of class				0.878	0.349
Yes	1589 (49.7%)	869 (48.93%)	720 (50.60%)		
No	1610 (50.3%)	907 (51.07%)	703 (49.40%)		
Professional qualifications				52.978	<0.001
Junior professional title	1143 (35.7%)	652 (36.71%)	491 (34.50%)		
Intermediate professional title	1675 (52.4%)	853 (48.03%)	822 (57.77%)		
Senior professional title	381 (11.9%)	271 (15.26%)	110 (7.73%)		
BMI	23.23 ± 6.81	23.09 ± 8.34	23.39 ± 4.18	1.529	0.216
Perceived Stress	29.02 ± 4.94	26.49 ± 3.72	32.16 ± 4.44	1543.546	<0.001

Values expressed as no. (%) or mean ( ± standard deviation).

### Prevalence of anxiety in primary school teachers

Mild to severe anxiety was identified in 44.5%(1423/3199) of primary school teachers under the “Double Reduction” Policy, who were categorized under the anxiety group. In this group, 56.43%(803/1423) reported mild anxiety, 25.30%(360/1423) reported moderate anxiety, and 18.27% (260/1423) reported severe anxiety. See **Table 2**.

**Table 2:** Distribution of anxiety severity levels among Chinese primary school teachers (N = 3,199).

Anxiety severity	GAD-7 score	n	%
Minimal	0–4	1776	55.5
Mild	5–9	803	25.1
Moderate	10-14	360	11.3
Severe	≥15	260	8.1

### Comparison of stress correlates between participants with and without anxiety

Chi-squared tests and analysis of variance revealed that there were significant differences between anxiety and non-anxiety groups in terms of age, years of teaching, education, fertility situation, income satisfaction, professional qualifications and perceived stress(all p < 0.05). Specifically, primary school teachers with younger age, shorter years of teaching, higher education level, dissatisfaction with income, fertility situation of two children, intermediate professional title and higher perceived stress scores had more severe anxiety syndrome. See **Table 1**.

### Factors associated with anxiety

In the final binary logistic regression model, five variables remained significantly associated with anxiety: education level, income satisfaction, professional title, age, and perceived stress score (**Table 3**). Teachers with a master’s degree or above were nearly 2.8 times more likely to experience anxiety than those with a junior college education or below (OR = 2.781, 95% CI: 1.858–4.163, p < 0.001). Those reporting dissatisfaction with income had 1.487-fold higher odds of anxiety than their satisfied counterparts (OR = 1.487, 95% CI: 1.205–1.834, p < 0.001). Individuals with an intermediate professional title were also more likely to experience anxiety than those with a junior title (OR = 1.372, 95% CI: 1.084–1.738, p = 0.009). Age was inversely related to anxiety (OR = 0.979, 95% CI: 0.966–0.993, p = 0.003), indicating that each additional year of age was associated with a 2.1% decrease in the odds of anxiety. Perceived stress demonstrated a positive association (OR = 1.489, 95% CI: 1.443–1.538, p < 0.001), with each one-point increase corresponding to approximately a 49% higher odds of anxiety.

**Table 3:** Binary logistic regression analyses of correlates of anxiety in primary school teachers.

Variables	Variables	Wald χ^2^	*P*-value	OR	95% CI
Education	Junior college or less			1.000	
	Bachelor	0.001	0.980	1.004	0.743-1.356
	Master or above	24.697	**<0.001**	2.781	1.858-4.163
Income satisfaction	Yes			1.000	
	No	13.662	**<0.001**	1.487	1.205-1.834
Professional qualifications	Junior professional title			1.000	
	Intermediate professional title	6.908	**0.009**	1.372	1.084-1.738
	Senior professional title	3.664	0.056	0.684	0.463-1.009
Age		8.888	**0.003**	0.979	0.966-0.993
Perceived stress scores		597.231	**<0.001**	1.489	1.443-1.538

CI, confidence interval; OR, odds ratio. P-values in bold indicate statistical significance (P < 0.05).

## Discussion

To the best of our knowledge, this is the first study to explore the prevalence and related factors of anxiety in Chinese primary school teachers during the “Double Reduction” implementation. The main findings are as follows: (1) The prevalence of anxiety symptom was 44.5%, and the majority of the teachers experiencing anxiety reported mild symptoms. (2) teachers with higher education level, dissatisfaction with income, intermediate professional title, younger age, and higher perceived stress were more likely to experience anxiety. This study provides a comprehensive overview of teachers’ mental health status in the context of ongoing education reforms.

In the current study, we found that under the “Double Reduction” Policy, the prevalence of anxiety among primary school teachers was relatively high (44.5%), which is much higher than that of the general population (6.0-31.9%) (29, 30). Anxiety among teachers was already a problem in China before the implementation of the “Double Reduction” Policy (7), and our findings add to this literature by showing a high prevalence of anxiety symptoms among primary school teachers during the period of policy implementation. There were several possible explanations for this atmosphere. First, increased workload may be one possible contextual factor related to teachers’ anxiety during the implementation of the policy. Teachers are required to redesign curricula and teaching plans to align with the new policy requirements, potentially leading to additional stress and longer working hours. Moreover, the introduction of new evaluation standards or methods may add to teachers’ responsibilities, creating further pressure; this highlights that the implementation of the “Double Reduction” Policy has greatly changed the work and life of primary school teachers. Although workload changes were not directly measured in the present study, previous research has reported that Chinese primary school teachers experienced longer working hours and heavier workload after the enactment of the “Double Reduction” Policy, particularly because of after-school services and adjustments to teaching tasks (18). These policy-related demands may have contributed to greater occupational stress among teachers. Second, primary school teachers may experience anxiety due to a lack of clear guidance on how to implement the new policy effectively. The ambiguity surrounding new teaching expectations adds to their stress. According to Uncertainty Anxiety Theory, humans have a natural tendency to seek certainty and predictability. When faced with ambiguous situations, such as unclear roles or frequent policy changes, individuals may feel a loss of control, which can lead to increased anxiety (31, 32). Third, as teachers may lack access to adequate mental health support, anxiety and stress levels are expected to rise. Schools often focus on providing psychological services for students, but teachers’ mental well-being is overlooked (33). Therefore, a psychological support program for primary school teachers is urgently needed in the context of the “Double Reduction” Policy.

Perceived stress showed a strong and independent association with anxiety among primary school teachers, with each one-point increase in stress score corresponding to nearly a 49% higher likelihood of experiencing anxiety. According to the stress-appraisal-coping model, anxiety arises when individuals evaluate environmental demands as exceeding their coping resources (34). The rapid educational reforms and increased administrative workload under the “Double Reduction” Policy may have amplified teachers’ perceptions of uncontrollable stressors, thereby heightening anxiety levels. This finding is consistent with prior studies showing that perceived stress mediates the relationship between job demands and mental health (35, 36). Interventions should therefore focus on stress reduction and coping enhancement, such as mindfulness-based programs, cognitive-behavioral training, and institutional workload adjustments to mitigate stress accumulation.

We also found that age was negatively associated with anxiety, indicating that younger teachers were more vulnerable to anxiety symptoms. Early-career educators often lack classroom management experience, face lower job control, and experience uncertainty regarding career advancement and employment security (37). These stressors may contribute to increased emotional exhaustion and anxiety. Similar patterns have been reported in occupational health research, where younger workers demonstrate higher stress reactivity (38). Tailored mentorship programs, workload management, and professional support networks could enhance younger teachers’ resilience and sense of control. Future studies may explore whether self-efficacy and social support mediate the age-anxiety relationship.

Interestingly, higher educational attainment (Master or above) was associated with greater anxiety. This may reflect a mismatch between high professional expectations and limited opportunities for academic or career advancement within the current school system. Overqualification and unmet expectations are well-known predictors of psychological distress (39). Highly educated teachers may also shoulder more complex responsibilities, including administrative and research tasks, without commensurate recognition or reward. Furthermore, low income satisfaction also emerged as a significant predictor of anxiety, consistent with the effort-reward Imbalance model (40). When teachers perceive that their workload and dedication are not adequately compensated, chronic stress and emotional strain may develop. The “Double Reduction” Policy may have inadvertently increased teachers’ workloads—through additional lesson preparation, student counseling, and parent communication—without corresponding income growth. Finally, teachers with intermediate professional titles reported higher anxiety levels. This group typically faces dual pressures from career advancement and family responsibilities, a stage sometimes referred to as the “mid-career squeeze”. They often manage heavy teaching loads while striving to meet the demanding criteria for promotion, which may contribute to persistent stress and burnout (41).

The present findings have several important implications for both research and practice. From a practical perspective, the strong link between perceived stress and anxiety underscores the need for multi-level interventions that address not only teachers’ individual coping skills but also systemic stressors within the school environment. Educational administrators should consider implementing structured stress management and emotional regulation programs, such as mindfulness-based interventions or cognitive-behavioral workshops, while simultaneously optimizing workload distribution and improving communication channels between teachers and policymakers. Enhancing income satisfaction and ensuring fair performance evaluation could also mitigate anxiety arising from perceived effort-reward imbalance. Furthermore, targeted psychological support should be offered to younger and mid-career teachers, who appear particularly vulnerable to anxiety due to career uncertainty and promotion pressures.

From a research perspective, these results highlight the importance of examining perceived stress as a potential mediator linking work conditions to mental health outcomes among teachers. Longitudinal and intervention studies are warranted to clarify causal relationships and evaluate the effectiveness of stress-reduction and support-based programs. Future research should also consider the role of contextual and organizational factors, such as leadership style, collegial climate, and policy communication, in shaping teachers’ psychological responses to educational reforms. Integrating quantitative and qualitative approaches may elucidate how macro-level policy changes translate into individual psychological distress and resilience within the teaching profession.

The findings presented in this study have some limitations, which should be considered when interpreting the results. First, a cross-sectional design can only present data at a specific instance, therefore the study is unable to establish causal relationships. In addition, it is not possible to determine whether the anxiety symptoms observed in the study subjects were pre-existing or newly developed cases. Second, the present research study is that we collected data based on self-reports of the prevalence of anxiety, and we did not use clinical measures (such as DSM-5). Therefore, the findings should be interpreted as reflecting self-reported anxiety symptoms rather than clinically diagnosed anxiety disorders. In addition, self-reported measures may be subject to reporting bias, including recall bias and social desirability bias, which may have affected the accuracy of participants’ responses. Third, we did not investigate the mental health help-seeking behaviors of primary school teachers with anxiety symptoms, which can inform the mental health policy-making and planning in the context of “Double Reduction” Policy. Additionally, this study was conducted during a highly stressful period in human history, the COVID-19 pandemic, which may have influenced the prevalence estimates of anxiety symptoms and the observed associations by introducing additional contextual stress. This context may also limit the generalizability of our findings to non-pandemic periods. Despite its limitations, this study is one of the few to report on the prevalence of anxiety symptoms among primary school teachers under the “Double Reduction” Policy in China.

## Conclusions

Our findings reveal the importance of regular anxiety screening for teachers to provide timely support to those in need. Stress management training would help teachers effectively deal with anxiety risk factors and improve their mental health. Future studies should consider more robust strategies such as a longitudinal study design to identify factors causing teachers’ anxiety under the “Double Reduction” Policy in China.

## Data Availability

The raw data supporting the conclusions of this article will be made available by the authors, without undue reservation.
